# Does benefit justify research with children?

**DOI:** 10.1111/bioe.12385

**Published:** 2017-09-08

**Authors:** Ariella Binik

**Keywords:** benefits, children, harms, paediatric research, research ethics

## Abstract

The inclusion of children in research gives rise to a difficult ethical question: What justifies children's research participation and exposure to research risks when they cannot provide informed consent? This question arises out of the tension between the moral requirement to obtain a subject's informed consent for research participation, on the one hand, and the limited capacity of most children to provide informed consent, on the other. Most agree that children's participation in clinical research can be justified. But the ethical justification for exposing children to research risks in the absence of consent remains unclear. One prevalent group of arguments aims to justify children's risk exposure by appealing to the concept of benefit. I call these ‘benefit arguments’. Prominent versions of this argument defend the idea that broadening our understanding of the notion of benefit to include non‐medical benefits (such as the benefit of a moral education) helps to justify children's research participation. I argue that existing benefit arguments are not persuasive and raise problems with the strategy of appealing to broader notions of benefit to justify children's exposure to research risk.

Paediatric research promotes children's wellbeing and medical care. It also helps to improve the limited evidence base supporting the safety and efficacy of medical interventions used in practice. The success of research depends on children's participation in clinical trials, but this is complicated by children's limited ability to make informed decisions to accept the risks of research participation. The problem is that it is not clear what our moral obligations are to children in health research. Should parents, researchers, and ethics committees enrol children and expose them to research risks or protect them at the cost of scientific progress? Many agree that moral duties to both current and future generations permit the enrolment of children in some clinical trials, but the ethical justification for exposing children to risk in the absence of their informed consent remains unclear.

In the following, I examine a prevalent group of arguments that aim to justify children's inclusion in research by appealing to the notion of benefit. I call these ‘benefit arguments’. Benefit arguments stem from the idea that research risks can be justified, at least in part, by appealing to the direct medical benefit an experimental intervention offers to a child. Benefit arguments extend this idea by arguing that research participation offers broader kinds of non‐medical benefits (e.g. the benefit of a moral education) and that these non‐medical benefits justify children's exposure to research risks. I argue that two prominent benefit arguments are unsuccessful and that the strategy of identifying new forms of non‐medical benefit is of limited use in justifying children's exposure to research risks. This work aims to examine the role of benefit, which has not received sufficient attention in paediatric research ethics, and to clarify what is needed for a successful justification for exposing children to research risk.

## 1 THE PROBLEM

The ethical inclusion of children in health research is often thought to merit special attention because of children's limited ability to make informed decisions about research participation. When adults participate in clinical trials, their understanding and informed consent to participate helps to justify their exposure to research risks. But when children cannot provide informed consent, it is not clear whether and why their participation is permissible. Questions about the ethics of risk imposition without consent are not unique to research with children. They also arise in discussions about appropriate social responses to natural hazards (such as flood relief programs), and in ‘technological safety analysis’, which examines risks generated by human activities (e.g. the storage of radioactive waste or the regulation of transport safety).^1^ The challenge in research with children is perhaps best understood as a subset of a problem in the philosophy of risk.

The philosophy of risk focuses on the development of a moral justification for the imposition of risk. A difficulty in this area is that in spite of ethical theories’ central concern with harming others and on justifications for imposing harm, they have devoted little attention to questions about the morality of imposing a risk of harm on others.^2^ Moreover, traditional moral theories struggle to accommodate the ethics of risk. Hayenhjelm and Wolff point out that consequentialism appears to be too permissive. It allows any risk in which benefits outweigh the costs, which oversimplifies difficult cases in which serious risks are outweighed by small aggregative benefits to many.^3^ Rights‐based theories also struggle with risk. In rights theories, harm is often thought to be mitigated by consent. Consent transforms a potentially rights‐violating act into a permissible act.^4^ But consent is unable to play the same role in justifying a risk of harm. Relying on consent as the justification for a risk of harm leads to what Hayenjhelm and Wolff call ‘The Problem of Paralysis’.^5^ The problem of paralysis is that most actions are accompanied by some amount of risk (however small) of significant harm to others. If risk imposition without consent is unjustifiable, then most actions are ethically impermissible.^6^ But this is an impossible position. To resolve the problem of paralysis, it is important to clarify what kinds of risk imposition do not violate individual rights and how they can be justified in the absence of consent.^7^

With respect to research with children, the problem is similar. Consequentialist reasoning appears to privilege the social value of health research for future children. But this may allow clinical trials to expose some children to high risks for the benefit of others, which seems to violate moral duties to particular children. Rights based theories offer a justification for research with competent adults. They justify a competent adult's risk exposure on the basis of her informed consent to accept the risk of harm. But when children (or adults) cannot provide informed consent, no such justification is available. Concluding that risk imposition without the child's consent is impermissible for children is both counter‐intuitive and undesirable, but more work is needed to establish what risks should be permitted and why they are permissible.

The problem of exposing children to research risk without consent is often thought to be especially pressing for particular kinds of research – clinical trials that do not offer direct medical benefits to the child participants themselves, but seek to generate scientific knowledge that will benefit future children. For instance, a trial might propose to perform serial bone marrow aspirates in children with acute lymphoblastic leukaemia to develop a better understanding of the suppression and re‐population of bone marrow.^8^ The bone marrow aspirates aim to provide scientific knowledge, but have no expectation of benefiting the children in the trial. This research, known as non‐therapeutic research, non‐beneficial research, or research that does not aim to offer children the prospect of direct medical benefit, is often thought to be especially ethically challenging because of the absence of both informed consent and medical benefit. Paul Ramsey – perhaps the most prominent early commentator on paediatric research – argued that non‐therapeutic research is unjustifiable. On his view, experimenting on children ‘in ways that are not related to them as patients is already a sanitized form of barbarism’.^9^

Further, even when research aims to benefit children medically, it involves interventions that are not in the medical interests of children, often called non‐therapeutic procedures. These procedures, such as additional blood draws for research purposes only, help to answer the scientific question but do not offer research subjects medical benefits. Given that non‐therapeutic procedures (within beneficial research) also expose children to research risk without corresponding medical benefits, they are also ethically challenging.

To summarize, the problem of justifying risk imposition without consent is a pressing problem for both moral theory and for the ethics of paediatric research. This problem seems to arise most urgently in the context of non‐therapeutic research or non‐therapeutic research procedures.

## 2 BENEFIT ARGUMENTS AS A SOLUTION

Ramsey understood the challenge posed by non‐therapeutic risks to children as irresolvable, suggesting simply that researchers ‘sin bravely’ in medical experiments.^10^ But others aim to offer justifications for risk exposure that are not based on consent. A prominent group of arguments grounds the justification for children's risk exposure on the notion of benefit. I call these *Benefit Arguments*.

Benefit arguments rely on the idea that the benefits of research participation play a central role in the justification of a trial. When research aims to offer children medical benefits, the potential for corresponding medical benefit helps to offset the risks. The idea that medical benefits offset risk is derived, in part, from an insight about the medical care of children. The insight is that the medical treatment of children involves risk, but this risk is justified when it is outweighed by the medical benefit for the child. This insight is ethically unproblematic. With respect to research with children, the idea is that in much the same way as in medical care, research offering the prospect of medical benefit can be justified by appealing to the medical benefits children might derive from participating in the trial. More generally, the idea is that medically beneficial research with children is less challenging because the research risks can be offset by the medical benefit. This is implicit in the special concern afforded to non‐therapeutic research.

Building on the significance of medical benefit in the justification for research risks, commentators argue that non‐therapeutic research risks can be justified if they are found to offer different, broader sorts of benefits. They then identify and defend different, non‐medical benefits that a child might derive from research participation. These are also *benefit arguments*. In what follows, I consider two benefit arguments appealing to non‐medical benefits to justify research with children. I then raise concerns for the argumentative strategy of appealing to benefits to justify children's exposure to research risks.

### 2.1 Benefit argument 1: Moral education

To help resolve the problem of justifying children's research participation in the absence of informed consent, some benefit arguments aim to identify broader, non‐medical kinds of benefits that justify a child's exposure to research risks. One such argument appeals to moral education as the broader non‐medical benefit. Proponents of this benefit argument claim that participation in research contributes to children's moral education and that this educational benefit justifies their exposure to research risk.^11^

One instance of this idea can be found in Terrence Ackerman's argument that parents are obligated to contribute to a child's moral development (Ackerman, 1979; Ackerman, 1980). Ackerman argues that parents must enhance the development of a child, which involves “inculcating interests and dispositions of character in him which will allow him to become a morally good, well‐adjusted and self‐fulfilled adult – a ‘right kind of person’” (Ackerman, 1979). For Ackerman, enrolment in non‐therapeutic research is one way in which parents may fulfil this obligation.^12^

Others have extended this idea to suggest that the benefit of a moral education justifies children's inclusion in clinical trials.^13^ For instance, Gaylin describes an instance in which a non‐therapeutic blood sample is requested from a child to further epidemiological research. The child initially refuses, but then complies at his father's insistence.^14^ Gaylin interprets the father's reasoning as suggesting the desire to teach his child a moral lesson – one ought to do certain things for the benefit of others even if they involve a small amount of pain.^15^ Non‐therapeutic research is one way in which parents can discharge their duty to teach children to be altruistic. On this view, children benefit by receiving a moral education that counterbalances research risk.

Redmon elaborates by suggesting that research may benefit a child by teaching her how one aspect of science works or by instilling in her a sense of pride in her contribution.^16^ Bartholome agrees, arguing that research participation may help children to ‘become sensitive to moral obligations’; it can help to foster in children a disposition towards choosing that which is good.^17^ These arguments conclude that research participation is one way in which to teach children to become benevolent people and that children's participation in some research is justifiable because it provides them with this educational – if not medical – benefit.^18^

While it seems reasonable to conclude that some children may derive educational benefit from research participation, this benefit argument encounters several problems. David Wendler points out that teaching children to be moral through research participation may well help them to become better members of society and to improve society overall, but that this seems to be a benefit to society rather than to the child herself.^19^ It follows that the educational value derived from participation is not well‐placed to justify a child's risk exposure. If the goal of moral education is to teach children to value being moral (rather than contributing to a better society), then it is not clear that research participation is a straightforward way to achieve this. This is because child research subjects experience the pain (e.g. needle jab) of research participation while broader societal benefits are often not realized for years. Activities in which the connection between the effort and benefit seems clearer seem better suited for teaching children to be altruistic and to value altruism.^20^ It follows that there are good reasons to doubt the success of arguments aiming to establish an educational benefit from research participation.

Brostrӧm and Johansson extend the critique further. They identify the conditions necessary for an argument to establish moral development as a benefit of research participation and argue persuasively that current versions fall short.^21^ They point out that a central weakness in these arguments is that at best, they can establish that children *may* derive the benefit of a moral education from research participation, but that the ‘*mere possibility*’ of benefit is insufficient.^22^ A successful justification depends on establishing a *significant likelihood* that children will receive this benefit but there are good reasons to doubt that this benefit will accompany participation for most children. For instance, they argue that for this benefit to be realistic, the moral education cannot be a by‐product of participation. It must be a significant goal of the research. Further, the particular educational benefit intended is not clearly articulated and if it were specified further, the scope of research likely to contribute to this goal would be rendered fairly narrow.^23^

Perhaps the most significant problem with this benefit argument is that it excludes a number of children from research participation entirely. Any child lacking the cognitive capacities necessary to learn the value of altruism is unable to benefit from research participation. It follows that she is an inappropriate research subject.^24^ But this means that infants and some young children cannot be research participants. Consequently, expanding the concept of benefit to include educational benefits does not provide a justification for research with all children. It follows that broadening the concept of benefit to include the benefit of a moral education is not sufficient to justify paediatric research.

### 2.2 Benefit argument 2: Physical contributions to a valuable project

David Wendler proposes a different benefit to justify children's exposure to research risk – the benefit of having contributed to a valuable project.^25^ The argument is that contributions to a valuable project offer an overall improvement to children's lives, even when the children are too young to take moral responsibility for their actions. It follows that even non‐autonomous, physical contributions to valuable projects such as clinical research benefit children by improving their lives.

This argument is derived from an account of human wellbeing comprised of five interests. The force of the argument is derived from the fifth interest, human achievements, which is defined as ‘accomplishments and contributions that are valuable for us given the kinds of being we are’.^26^ The idea is that a good life involves good accomplishments. The innovation is the suggestion that worthwhile projects benefit people in more ways than are generally recognized. Most think the value derived from a person's participation in a worthwhile project is derived from the active contribution made to that project. For instance, the value of helping to build homes for the homeless is usually thought to rely on understanding the project, embracing its goals, and contributing in some way to its realization (perhaps by fundraising or locating a construction site). According to this interpretation, only autonomous people can derive value from participation in valuable projects.^27^ But the argument endorses a broader understanding of benefit according to which the ‘mere fact of being part of a worthwhile effort is valuable’.^28^ If involvement in worthwhile projects is valuable in and of itself, it follows that contributions to worthwhile projects – such as clinical trials – can improve children's lives even when they are unable to take moral responsibility for their participation.^29^

To support the argument, we are asked to consider examples of what constitutes a good or bad life for a child. In one example, we are asked to consider two possible lives for a child. In Life A, a toddler accidentally shoots and kills a playmate. In Life B, the child lives a similar life without this accident. Wendler argues that Life B would be preferred by any parent, even if the child does not understand the accident or come to remember and regret it later in life. The rationale is that having killed an innocent person is bad for a child. It thwarts the child's interests, irrespective of the child's experiences or preferences. This example (Accident) aims to show that physical involvement in negative projects (such as accidental shootings) make a life worse by becoming part of the life's narrative, which then influences its overall quality.^30^

The argument from Accident is then extended to suggest that physical contributions to valuable projects can improve a life. Consider, for example, a child's participation in a clinical trial aiming to develop a vaccine for rotavirus. Rotavirus – a common virus – kills hundreds of thousands of children every year, primarily in resource scarce settings. To help save children's lives and to reduce the burdens of a disease felt disproportionately in low‐income countries, a research programme was undertaken that subsequently led to the development of two safe and effective vaccines for rotavirus.^31^ This is an instance of significantly valuable research that depended, in part, on children's participation in clinical trials and exposure to some research risks that are not in their direct medical interests.

To support the benefit argument concerning physical contributions to a valuable project, we are asked to consider the same thought experiment: Put yourself in the place of a parent considering two possible lives for a child. In Life A, the child has a good and decent life and in Life B, the child has the same life but also participates in one of the trials leading to a vaccine for rotavirus.^32^ The child is too young to understand, appreciate, or consent for her involvement. Wendler argues that when evaluating the quality of two similar lives, Life B is preferable because the non‐autonomous contribution becomes part of a positive narrative that improves a life overall. This argument suggests that children's exposure to research risks is justifiable because it improves a child's life by providing her with a contribution to a valuable project.

This is the most robust benefit argument, but it is subject to limitations. One problem is that intuitions captured by Accident and Vaccine can be accommodated without appealing to the idea that physical contributions to valuable projects are in the interests of children. These examples are perhaps better explained by appealing to parents’ preferences for their children. Consider the argument supporting Accident. The idea is that a child's life would be better if it did not include the accidental killing of a playmate even if the child was too young to understand the accident, was not negatively affected, and never came to regret the action. Wendler argues that the reason this seems plausible is not related to a child's experiences (she doesn't remember) or her preferences (she is too young to have autonomous preferences). Instead, the rationale is that it is better for children, for their own sakes, that they never be the cause of such an accident. To support this view, he points out that parents do not remain neutral about Accident. Wendler writes:

Try to imagine a caring and reasonable parent thinking as follows: ‘Well, if my child is not harmed in any way and does not come to regret it, then, as far as my child is concerned, it does not matter whether his friend dies as the result of an earthquake or dies as the result of my child shooting him.’ To my mind at least this is unimaginable.^33^

It is clear that most would prefer that a child's life does not involve an unintentional killing. But it is less clear that the reason this is preferred is because it is in the interests of the child. Perhaps the most plausible reason Accident is undesirable is because it thwarts a parent's preference for a child's life. Most parents desire lives for their children that involve happy, worthwhile events. They also aim to protect children from events that will compromise their current interests or actions that are likely to compromise their future preferences. Perhaps the most plausible understanding of the undesirability of Accident is not that it is bad for the child for her own sake, but that it compromises a parent's preferences for her child's life or reflects a parent's reasonable concern that the child will ultimately come to learn about and regret the unintentional shooting.

Another example may help to illustrate. Suppose that a sleeping infant carried by her parent is photographed during a ‘Take Back the Night’ rally protesting violence against women. The photograph appears on the front page of a newspaper, helps to generate public sympathy, and contributes to the rally's success. But the infant's parent never notices the photograph ‐ she was simply crossing the street at an opportune moment – and the infant never learns about her passive contribution to the valuable project. There are good reasons to prefer a world in which this rally achieves its goals. But there is no clear reason to think that a child's physical contribution to this rally has any bearing on the overall quality of that child's life. Any benefit to a child from Rally seems to depend on a parent's intent, preference, or knowledge of the child's involvement or the child's later knowledge of and embracing the involvement. That is to say, in the absence of the child or parent's intent, preference, or knowledge of the physical contribution to a valuable cause, there is no reason to think this involvement in a valuable project has any bearing on the overall quality of that child's life. The negative impact of Accident and the positive impacts of Vaccine and Rally seem to be derived from the effect they have on parents’ preferences or on a parent's expected preferences for the child's future, which challenges the suggestion that non‐autonomous contributions to valuable projects impact a child's life.

Perhaps a more pressing limitation to this argument is that even if we accept that there are a list of interests that contribute to a child living a good life, there are good reasons to question whether human achievements should appear on this list. Commonly held moral intuitions do not support the idea that physical, non‐autonomous contributions to a valuable project improve a child's life when that child does not come to embrace these contributions later in life.^34^

The example of benefits conferred through participation in a rotavirus vaccine trial seems plausible, but once we consider trials with inconclusive or negative outcomes (which account for a significant number of clinical trials), the argument is less plausible. Consider ‘Hypothermia’, a study that examined the efficacy of hypothermia therapy in children who have suffered traumatic brain injury.^35^ At the time the study was developed, hypothermia therapy had shown significant improvement in survival and neurological outcomes in rodent models. To examine whether hypothermia therapy could also help children with brain injuries, researchers conducted a randomized controlled trial including 255 children aiming to establish whether hypothermia therapy started within 8 hours of injury may improve outcomes in children.^36^ This trial found that hypothermia therapy does not improve the neurological outcome and may increase mortality in children with severe traumatic brain injury.^37^

There is little question that this trial is valuable. It offers some evidence that in spite of success in animal models and limited evidence from trials with small sample groups, hypothermia therapy may not be a useful intervention for children with brain injuries. These results may become instrumental in the improved medical treatment of other children. More generally, negative trials are valuable projects. Knowledge derived from them may help to reduce harm by stopping treatments that don't work and may help to encourage new therapeutic options.^38^

The problem raised by this example is that once we consider negative trials (in place of trials with positive outcomes such as the Rotavirus example), they challenge the intuition that physical participation in valuable clinical trials necessarily contributes to a better life. That is, analysed according to Wendler's thought experiment, Hypothermia does not clearly support the idea that contributions to valuable projects improve the child's life. If we put ourselves in the shoes of a parent considering two possible lives for a child – Life A, in which a child participates in the hypothermia therapy trial or Life B, in which the child does not – there is no obvious reason to prefer the life involving participation in Hypothermia. The trial is an instance of a valuable project that derived knowledge about the limited success of hypothermia therapy. But it also involved a negative result. And when considering valuable clinical trials that yield negative results, it is far more likely that intuitions will vary about whether a life that includes participation in research is preferable. That is, there is no obvious reason to think non‐autonomous participation in a trial that derived valuable knowledge but did not benefit and may have harmed child participants is preferable to a life that involved no research participation.^39^

This suggests that physical contributions to a valuable project do not offer children the benefit of an improved life. To summarize, examples offered in support of this benefit argument do not seem to support the idea that physical contributions to a valuable cause contribute to a better life for a child overall.

## 3 THE STRATEGY

Where does this leave benefit arguments? I've argued that including children in research is valuable, but difficult to justify. The problem is that paediatric research imposes risks of harm on children and these risks cannot be justified by the child's informed consent. Consequently, the ethical justification for children's inclusion in research depends on a different justification for risk exposure. Some benefit arguments aim to meet this challenge by identifying broad, non‐medical benefits that accrue to children's research participation. Two such arguments—appealing to the benefit of a moral education or physical contributions to a valuable project—are not persuasive. But will a different benefit argument solve the problem? Examining the argumentative strategy suggests that identifying broader forms of benefit cannot successfully justify children's risk exposure.

Benefit arguments are based on the idea that the absence of informed consent complicates children's inclusion in research. But that when research offers the prospect of medical benefit, it is less ethically contentious. They then extend this idea by identifying non‐medical benefits that children can derive from research participation. These broader benefits aim to reduce the contentiousness of non‐therapeutic research. Benefit arguments take the following form:

P1: When children cannot provide informed consent, it is difficult to justify their exposure to research risks

P2: When research offers children the prospect of direct medical benefit, it is less ethically challenging

P3: Identifying a different, broader, non‐medical benefit that accrues to research participation helps to justify children's exposure to non‐therapeutic research risks

P4: Research offers children the benefit of X (where X is a non‐medical benefit)

C1: X offsets the non‐therapeutic research risks (from P3 and P4)

C2: Research (or research interventions) without medical benefit are justifiable because they offer children the benefit of X (from P2, P3, P4, and C1)

In the sections above, I challenge the idea that moral education or physical contributions to a valuable project are benefits that children derive from research participation (i.e. challenges to P4). But premises 2 and 3 also merit careful attention. Clarifying these premises reveals a problem with the argumentative strategy, which suggests that attempts to identify a different form of benefit are unlikely to justify paediatric research.

The idea that research offering children medical benefit is less ethically challenging (P2) is a prevalent and longstanding belief in paediatric research ethics. It derives its force from the idea that when a child is exposed to the risks of an experimental intervention, the possibility of a corresponding medical benefit helps to justify the risks. For instance, the risk of an experimental chemotherapy drug may be balanced by the potential for this drug to achieve remission. This is plausible. The potential for medical benefit contributes to the justification of the risks imposed by a research intervention. But I would like to suggest that the role of benefit in justifying paediatric research has been over‐extended and under‐explored. The problem is that benefit arguments privilege the identification of sources of benefit without paying sufficient attention to the role this benefit plays in justifying a trial. An examination of the role of benefit finds that identifying non‐medical benefit has a limited purview in the ethical justification for children's exposure to research risk.

What is the role of benefit in the broader justification for paediatric research? Early commentary reflects the view that research offering children medical benefit is ethically unproblematic. Discussion focused instead on the ethics of research that is not in a child's medical interests. For example, Henry Beecher writes that ‘[w]hen experimentation in children is for diagnosis or treatment for the direct benefit of the child, the ethical problems are few as long as the consent of the parent or guardian has been obtained’.^40^ Similarly, William May claims that ‘[t]here is no serious debate among authorities, medical, legal, or moral, when the experiment in question is therapeutic…*there is unanimity that in therapeutic situations such proxy consent is morally justifiable*’.^41^ This suggests that in early commentary, benefit played the central role in justifying children's exposure to research risks. If a trial offered children medical benefit, then it was justifiable. Non‐therapeutic research with children was the central cause for concern.

These views shaped the subsequent debate over research with children, but the idea that beneficial research is ethically unproblematic is no longer tenable. Early commentaries are based on the idea that research can be strictly divided into two categories: therapeutic research, which offers medical benefit to the research subjects themselves and non‐therapeutic research, which is designed to further biomedical and behavioural research. These views are problematic because no strict distinction can be drawn between therapeutic and non‐therapeutic research.^42^ Most clinical research involves a combination of procedures, some of which are administered with therapeutic warrant and some of which aim to answer the study question (sometimes known as therapeutic and non‐therapeutic procedures).^43^

Moreover, the early concern with non‐therapeutic research also relies on the belief that some research is exclusively in the interests of children. This is untenable because all clinical research that offers children the prospect of medical benefit also contains non‐therapeutic procedures. For example, a study aiming to evaluate the efficacy of an experimental drug for the alleviation of pain in children with cancer also involves non‐therapeutic procedures, such as the review of medical records or radiological investigations that have no bearing on clinical care.^44^ For these reasons, the distinction between therapeutic and non‐therapeutic research is no longer endorsed.^45^

What are the implications of this for paediatric research? Recognizing that research offering the prospect of benefit also involves non‐therapeutic procedures suggests that beneficial research is not ethically unchallenging. That is, clinical trials are not rendered uncontroversial by virtue of offering the prospect of direct medical benefit. Some trials that offer medical benefit are ethically impermissible. For example, a trial offering research subjects the prospect of medical benefit that is significantly lower than that which they would receive during the course of standard care unfairly deprives children of competent medical care. In addition, a trial offering child research subjects the prospect of some medical benefit in addition to highly invasive interventions administered without therapeutic warrant is probably impermissible. In spite of the benefit, it would expose children to high degrees of research risk purely in the interest of others. More generally, the suggestion is that the ethical permissibility of a trial depends on more than benefit; the morally relevant consideration is not whether a trial offers benefit but whether the potential benefit of a trial stands in reasonable relation to the research risks.

The idea that the ethical justification for children's risk exposure depends on an appropriate balance between harms and benefits (rather than benefit alone) is well reflected in current ethics guidelines and commentary.^46^ For instance, Wendler's argument about children's contributions to valuable projects is constrained according to additional conditions, including a ‘risk ceiling’ and ‘risk allowance’ condition, which aim to limit the amount of risk permitted in research with children. He also writes that enrolling children is consistent with their interests only ‘when the risk/benefit ratio of the research is at least as favourable as that of available alternatives’.^47^ Further, he suggests that physical contributions to a valuable project can only lead to a marginally better life for a child. And given that these contributions only benefit children to a minor extent, they can only justify exposing children to low risks.^48^ Similarly, Bartholome's argument about the benefit of a moral education are meant to justify only research involving ‘no greater risk or discomfort than would be encountered by the child in his family life’.^49^ This is perhaps best understood as suggesting that arguments no longer rely uniquely on the identification of benefit to justify paediatric research. They rely on a balance between risks and potential benefits. That is, determinations about the permissibility of research depend on the potential benefits offsetting the risks of participation.

Premise 2 of the benefit arguments – the idea that beneficial research is less ethically challenging – is consistent with the idea that benefit alone does not justify paediatric research. The problem is that the significance of identifying new sources of benefit has been over‐extended. Premise 3 claims that the new, non‐medical benefits of research participation help to justify children's participation in research that offers no corresponding medical benefit. But this merits careful consideration. Clarifying the idea that beneficial research is less ethically challenging (P2) reveals that potential medical benefits may be used to help offset the risks of experimental interventions, but only when they are part of a favourable risk‐benefit ratio. In other words, the role of benefit in justifying children's exposure to research risk is constrained. It follows that identifying broad, non‐medical benefits to research participation should be subject to the same constraints. Any non‐medical benefits must stand in reasonable relation to the risks of the trial.

Recognizing a constrained role for non‐medical benefits in the ethical justification for paediatric research raises difficult questions about the purview of benefit arguments. The new benefits identified in these arguments aim to show that even non‐therapeutic research (or non‐therapeutic research procedures) offer children some sort of benefit. But it is not clear whether and why this new form of benefit justifies any particular risks or how it contributes to a risk‐benefit calculus.

Current commentary and guidelines agree that both beneficial and non‐beneficial research with children should be subject to constraints. For instance, non‐beneficial research (or research interventions) are often constrained according to a minimal risk threshold.^50^ Similarly, beneficial research is also constrained. It is often thought to be permissible only when it is at least as good as available alternative.^51^ The problem is that it is far from clear how new non‐medical benefits should be incorporated into these calculations and balanced against the existing risk thresholds. Given that the new benefits are meant to attach to research or research interventions that do not aim to offer the prospect of medical benefit, one possibility is that they should be subject to the same constraints as other non‐beneficial risks. If the new forms of benefit should be subject to the constraints of non‐beneficial risks, they should be measured according to the minimal risk threshold (or some other threshold for maximal allowable risk without benefit). But if this is the case, then it is the upper threshold for allowable risk justifying children's risk exposure and not the broader non‐medical benefits.

Another possibility is that determining that non‐therapeutic risks offer some broader non‐medical benefits means that these procedures (or research) should be recognized as beneficial after all. If this is the case, they must be subject to another set of constraints – that the procedures are at least as beneficial as available alternatives. But understood in this way, one is left with an implausible scenario in which non‐medical benefits attached to research procedures are weighed against alternative medical treatments for diseases and disorders of childhood. For instance, it is far from clear how accepting the idea that non‐therapeutic lumbar punctures for children with cystic fibrosis pose educational benefits might factor into an analysis of whether an educationally beneficial lumbar puncture is as beneficial as alternative medical options for cystic fibrosis. More generally, it is difficult to understand how identifying broader non‐medical benefits to research participation contributes to the risk‐benefit ratio of a clinical trial. It follows that there is no straightforward means by which broader, non‐medical benefits offset non‐therapeutic research risks.

Incorporating non‐medical benefits into risk‐benefit calculations raises a number of difficult questions. For instance, should non‐medical benefits of research participation be balanced against the overall value of a trial? Do they offset only the risks of research procedures that offer no corresponding medical benefit? Are there limits to the amount of non‐medical benefit that may justify particular research risks? How should a non‐medical benefit be balanced against a non‐therapeutic research procedure? That is, how should the potential benefit of a moral education be balanced against the risks of a non‐therapeutic lumbar puncture? Does it pose less than minimal risk? Should it be compared with other beneficial interventions? Without answers to these questions, the conclusions of the benefit argument do not follow. That is, it is not clear why or how a non‐medical benefit offsets the risks of non‐beneficial research (C1) and consequently, these benefits do not justify children's research participation.

More generally, emphasizing the identification of new sources of benefit risks extending the purview of benefit in the ethics of research with children by shifting focus away from risk‐benefit determinations. It follows that a main task of benefit arguments is to elaborate on how a new source of benefit should be factored into risk‐benefit determinations to contribute to a persuasive justification for children's inclusion in research. Ultimately, the success of identifying new forms of benefit depends on further attention to the role of these benefits in justifying children's exposure to risk.

## 4 CONCLUSION

The progress of children's medical care depends on their inclusion in clinical trials, but the ethical inclusion of children in research is complicated by their limited capacity to provide informed consent to undertake the risks of research. The ethical justification for risk exposure in the absence of consent is a broad problem not only for the ethics of research with children but also for research with adults lacking capacity, for developing and implementing public policies, and for the philosophy of risk more broadly. The challenge is to explain what legitimizes a risk of harm, rather than the imposition of a harm itself. Prominent moral theories do not seem to offer straightforward solutions to questions about risk imposition without consent. But the ethical inclusion of children in research depends on resolving this tension.

A group of arguments aim to justify children's exposure to research risk by appealing to the notion of benefit. These arguments stem from the idea that the potential for direct medical benefit helps to justify a child's exposure to experimental research interventions. This is a long‐standing and prominent strategy for the justification of paediatric research. This idea is then extended by identifying new forms of non‐medical benefit that aim to justify children's inclusion in clinical trials. I have argued that identifying moral education or physical contributions to a valuable project as benefits of research participation are unpersuasive and I have raised limitations to the argumentative strategy of benefit arguments. In conclusion, the success of benefit arguments depends on further attention to the role of new sources of benefit in risk‐benefit determinations rather than on the identification of new sources of non‐medical benefit.

## Footnotes

1
Hayenhjelm, M & Wolff, J. (2011). The moral problem of risk impositions: a survey of the literature. *European Journal of Philosophy*, *20*(S1), e26–e51.

2
Ibid; Hansson, S.O. (2013). *The Ethics of Risk: Ethical Analysis in an Uncertain World* London: Palgrave MacMillan; Teuber, A. (1990). Justifying risk. *Daedalus 119*(4), 235–254; Lewens, T. (2007). *Risk: Philosophical Perspectives*. London and New York: Routledge.

3
Hayenhjelm & Wolff, *op. cit.* note 1.

4
Teuber, *op. cit.* note 2.

5
Hayenhjelm & Wolff, *op. cit.* note 1.

6
Ibid.

7
Ibid.

8
National Human Research Protections Advisory Committee (NHRPAC). (2002). *Clarifying Specific Portion of 45 CFR 46 Subpart D that Governs Children's Research* NHRPAC. Retrieved from https://ctep.cancer.gov/investigatorResources/childhood_cancer/docs/nhrpac16.pdf

9
Ramsey, P. (1976). The enforcement of morals: Nontherapeutic research on children. *Hastings Center Report 6*(4), 21–30. 133992

10
Ibid.

11
Beecher, H. K. (1970). *Research and the Individual: Human Studies* Boston, MA: Little, Brown and Company; Bartholome. W. G. (1976). Parents, children, and the moral benefits of research. *Hastings Center Report 6*(6), 44–45; Ackerman, T. F. (1979). Fooling ourselves with child autonomy and assent in nontherapeutic clinical research. *Clinical Research, 27*(5), 345–348; Ackerman, T. F. (1980). Moral duties of parents and nontherapeutic clinical research procedures involving children. *Bioethics Quarterly*, *2*(2), 94–111; Gaylin, W. (1982).The competence of children: no longer all or none. *The Hastings Centre Report*, *12*(2), 33–38; Redmon, R. (1986). How children can be respected as ‘ends’ yet still be used as subjects in non‐therapeutic research. *Journal of Medical Ethics, 12*, 77–82.

12
On Ackerman's account, parents are morally obligated to contribute to children's moral development, but the benefit of a moral development does not necessarily legitimize children's participation in nontherapeutic research. Nontherapeutic research with children is permissible only if it falls below an upper threshold for permissible risk – the risks involved in family life (Ackerman, 1980, *op. cit.* note 11).

13
See Brostrom and Johansson, 2014, p. 54 for a comprehensive summary and analysis of contributions to the moral development argument. Brostrӧm, L & Johansson, M. (2014). Involving children in non‐therapeutic research: on the development argument. *Medicine, Healthcare and Philosophy*, *17*, 53–60. 10.1007/s11019-013-9486-023690163

14
Gaylin, *op. cit.* note 11, p. 37.

15
Ibid.

16
Redmon, *op. cit.* note 11.

17
Bartholome, *op. cit.* note 11, p. 17.

18
See Lainie Friedman Ross for another argument about how research participation contributes to a child's moral education. This argument differs in that the main focus is on parental discretion in determining what activities are permissible for children. On Ross's argument, parental authority for enrolling children in research without benefit is constrained to minimal risk research. Ross, L. F. (1998). *Children, Families, and Health Care Decision Making*. Oxford: Clarendon Press.

19
Wendler, D. (2010). *The Ethics of Pediatirc Research* (p. 93). Oxford: Oxford University Press.

20
Ibid: 94.

21
Brostrӧm & Johansson, *op. cit.* note 13.

22
Ibid.

23
Ibid.

24
Ackerman, *op. cit.* note 11.

25
Wendler, *op. cit.* note 19; Wendler, D. (2012). A new justification for pediatric research without the potential for clinical benefit. *American Journal of Bioethics, 12*(1), 23–31. 10.1080/15265161.2011.63448222220955

26
Wendler, *op. cit.* note 19, p. 133.

27
Ibid.

28
Ibid, p. 130.

29
Wendler, *op. cit.* note 19; Wendler, *op. cit.* note 25.

30
Ibid.

31
Wendler, *op. cit.* note 25.

32
Ibid.

33
Ibid: 27.

34
The suggestion in this section is not that children's welfare cannot be accounted for by appealing to a list of things that are in their interests. Elsewhere, I've defended an objective list account of children's welfare for different reasons (Binik, A. (2014). On the Minimal Risk Threshold in Research with Children. *The American Journal of Bioethics*, *39*(4), 387–405). Instead, the suggestion is that the examples offered are at least equally well supported by parental preference, which challenges the idea that human achievements should be recognized as contributing to children's welfare.

35
Hutchison, J. S., Ward, R. E., Lacroix, J. Hébert, P., Barnes, M., Bohn, D. J….Skippen, P. W. (2008). Hypothermia therapy after traumatic brain injury in children. *The New England Journal of Medicine*, *358*(23), 2447–2456. 10.1056/NEJMoa070693018525042

36
Ibid: 2448.

37
Ibid: 2454.

38
Robert Wachbroit objects to Wendler's benefit argument by pointing out that at the beginning of an experiment, one cannot be certain of its success and that when experiments turn out to be unsuccessful (failed or negative hypothesis), it is implausible to think that participation in them is a valuable project. He concludes that a justification for pediatric research based on benefit derived from participation in valuable projects does not include most clinical research. See Wachbroit, R. (2012). On Wendler's new justification for pediatric research. *The American Journal of Bioethics*, *12*(1), 40–42. The current argument accepts that negative trials are valuable, but questions the idea that participation in this sort of valuable activity improves the quality of a child's life.

39
See Peter Singer for another objection to the idea that children can benefit or be harmed by contributions to projects made before they can take morally responsibility for their contribution. Singer, P. (2011). When is research on children ethical? *The Lancet*, *377* (9760), 115–116.

40
Beecher, *op. cit*. note 11, p. 63.

41
May, W. E. (1977). *Experimenting on Human Subjects. Existence, Medicine, and Ethics* (p. 21). Chicago: Franciscan Herald Press, emphasis added.

42
Levine, R. (1986). *Ethics and Regulation of Clinical Research* (2nd ed., pp. 8–9). New Haven, London: Yale University Press; Levine, R. (1999). The need to revise the declaration of Helsinki. *The New England Journal of Medicine 341*(7), 531–534. 10.1056/NEJM19990812341071310441613

43
Levine (1986), *op. cit.* note 38; B. Freedman, B., Fuks, A. & Weijer, C. (1992). Demarcating research and treatment: a systematic approach for the analysis of the ethics of clinical research. *Clinical Research, 40*(4), 653–660. 1486732

44
Weijer, C & Miller, P. B. (2004). When are research risks reasonable in relation to anticipated benefits? *Nature Medicine, 10*, 570–573. 10.1038/nm0604-57015170195

45
The insight that all therapeutic research involves non‐therapeutic procedures has an important impact on Ramsey's justification for the inclusion of children in research that is in their interests. Ramsey's argument relies on the idea that some research is purely in the interests of children. It is permissible because it offers a child the best prospect for recovery and no aspect of it is administered to benefit other children. Research procedures that are not administered with therapeutic warrant are strictly prohibited; see Ramey, P., (1970). *The Patient as Person: Explorations in Medical Ethics*, Second edition. New Haven: Yale University Press.Thus, on Ramsey's view, non‐therapeutic research procedures – such as data collection and extra examinations – are impermissible for those who cannot provide informed consent. Given that all therapeutic research – including research that aims to offer the child subjects the prospect of direct benefit – includes both kinds of procedures, Ramsey's argument suggests that it is impermissible to involve children in any research, including beneficial research.

46
U.S. Department of Health and Human Services (HHS). (2009). 45 C.F.R. Part 46 Subpart D. Retrieved from https://www.hhs.gov/ohrp/regulations-and-policy/regulations/45-cfr-46/; Belmont Report. 1979. National Commission for the Protection of Human Subjects. The report to the Secretary of the Department of Health, Education and Welfare. Retrieved from https://www.hhs.gov/ohrp/regulations-and-policy/belmont-report/; Council for International Organizations of Medical Sciences (CIOMS) in collaboration with the World Health Organization (WHO). 2016. *International Ethical Guidelines for Health Related Research involving Humans* Geneva, Switzerland. Retrieved from http://cioms.ch/ethical-guidelines-2016/WEB-CIOMS-EthicalGuidelines.pdf

47
Wendler, *op. cit.* note 25, p.24.

48
Ibid: 29.

49
Bartholome, *op. cit.* note 11.

50
DHHS Regulations, *op. cit.* note 41; CIOMS, *op. cit.* note 41; L. Kopelman. (2204).Minimal risk as an international ethical standard in research. *Journal of Medicine and Philosophy*, *29*(3), 351–78; Wendler, *op. cit.* note 18; Ross, *op. cit.* note 17. 10.1080/0360531049050054515512977

51
DHHS Regulations, *op. cit.* note 41; CIOMS, *op. cit.* note 41; Weijer & Miller, *op. cit.* note 40; Ramsey, *op. cit.* note 9.

